# The kallikrein-kinin system in experimental Chagas disease: a paradigm to investigate the impact of inflammatory edema on GPCR-mediated pathways of host cell invasion by *Trypanosoma cruzi*

**DOI:** 10.3389/fimmu.2012.00396

**Published:** 2013-01-25

**Authors:** Julio Scharfstein, Daniele Andrade, Erik Svensjö, Ana Carolina Oliveira, Clarissa R. Nascimento

**Affiliations:** Laboratório de Imunologia Molecular, Instituto de Biofísica Carlos Chagas Filho, Centro de Ciências da Saúde, Universidade Federal do Rio de JaneiroRio de Janeiro, Brazil

**Keywords:** bradykinin, cardiomyopathy, cruzipain, endothelins, GPCRs, proteases, kallikrein, *Trypanosoma cruzi*

## Abstract

Chronic chagasic myocarditis (CCM) depends on *Trypanosoma cruzi* persistence in the myocardium. Studies of the proteolytic mechanisms governing host/parasite balance in peripheral sites of *T. cruzi* infection revealed that tissue culture trypomastigotes (TCTs) elicit inflammatory edema and stimulate protective type-1 effector T cells through the activation of the kallikrein-kinin system. Molecular studies linked the proinflammatory phenotype of Dm28c TCTs to the synergistic activities of tGPI, a lipid anchor that functions as a Toll-like receptor 2 (TLR2) ligand, and cruzipain, a kinin-releasing cysteine protease. Analysis of the dynamics of inflammation revealed that TCTs activate innate sentinel cells via TLR2, releasing CXC chemokines, which in turn evoke neutrophil/CXCR2-dependent extravasation of plasma proteins, including high molecular weight kininogen (HK), in parasite-laden tissues. Further downstream, TCTs process surface bound HK, liberating lysyl-BK (LBK), which then propagates inflammatory edema via signaling of endothelial G-protein-coupled bradykinin B_2_ receptors (BK_2_R). Dm28 TCTs take advantage of the transient availability of infection-promoting peptides (e.g., bradykinin and endothelins) in inflamed tissues to invade cardiovascular cells via interdependent signaling of BKRs and endothelin receptors (ETRs). Herein we present a space-filling model whereby ceramide-enriched endocytic vesicles generated by the sphingomyelinase pathway might incorporate BK_2_R and ETRs, which then trigger Ca^2+^-driven responses that optimize the housekeeping mechanism of plasma membrane repair from cell wounding. The hypothesis predicts that the NF-κB-inducible BKR (BK_1_R) may integrate the multimolecular signaling platforms forged by ceramide rafts, as the chronic myocarditis progresses. Exploited as gateways for parasite invasion, BK_2_R, BK_1_R, ET_A_R, ET_B_R, and other G protein-coupled receptor partners may enable persistent myocardial parasitism in the edematous tissues at expense of adverse cardiac remodeling.

## INTRODUCTION

Afflicting nearly 10 million people in Latin America (Coura and Dias, 2009), Chagas disease is a pleiomorphic clinical entity caused by *Trypanosoma cruzi*, a parasitic protozoan that undergoes obligate intracellular development in the mammalian host. Extremely polymorphic (Macedo and Pena, 1998), the natural populations of *T. cruzi* have been recently subdivided into six discrete taxonomic units (DTUs) named *T. cruzi* I to *T. cruzi* VI (Zingales et al., 2009), of which at least four are known to be involved with human pathology (Miles et al., 2009). Whether transmitted to humans via mucosal wounds inflicted by hematophagous vectors of the reduviid family or, indirectly, by oral ingestion of contaminated juices (Coura and Dias, 2009; Cortez et al., 2012), the insect-derived infective forms (metacyclic trypomastigotes) induce an acute phase that may be asymptomatic, or life-threatening. Characterized by high blood parasitemia, the sequels of severe acute disease may include hepatosplenic pathology, myocarditis, and more rarely, encephalitis. Lasting a few months, the acute symptoms subside with the onset of immunity, but the effector response is not capable of eradicating the intracellular parasites, leading to a chronic infection, characterized by low-grade tissue parasitism and positive serology. Several years later, about 30% of the patients develop a full-blown chronic chagasic myocardiopathy (CCM), characterized by the presence of inflammatory T cell infiltrates, myocardial fibrosis, complex arrhythmias, thromboembolism, and ventricular aneurysms (Marin-Neto et al., 2007). Patients with severe forms of CCM may have heart failure and sudden death, while the remaining chagasic patients (indeterminate stage) remain asymptomatic for decades. In the south cone of America, chagasic patients may also develop digestive system abnormalities (megacolon and/or megaesophagus), albeit in lower frequency than CCM.

## CCM: CONVERGING PATHOGENIC MECHANISMS

Nearly a century after the discovery of Chagas disease, we have come to realize that the mechanisms responsible for the variable clinical manifestations during the chronic phase are still elusive. Cardiac parasympathetic depopulation, microvascular derangement, and low-grade myocardial inflammation directly induced by parasites and T cell-dependent immunopathology seem to converge in the genesis of CCM. After decades of debate, there are persuasive arguments supporting the notion that the primary cause of CCM is a low-grade, persistent parasitism of the myocardium (Tarleton, 2001). A large body of studies in mice and humans indicated that chronic myocarditis is critically dependent on the recruitment of parasite-specific (type 1) effector CD8 T cells to the infected cardiac tissues (Padilla et al., 2009; Silverio et al., 2012).

While not dismissing the relevance of intracardiac infiltrates in the progression of CCM, vascular pathologists argued that low-grade infection could lead to the accumulation of microvascular lesions in the chagasic heart, ultimately resulting in myocardial hypoxia, which in turn may aggravate collateral injury inflicted by pathogenic T cells infiltrating the heart (Morris et al., 1990; Rossi, 1990; Higuchi et al., 1999, 2003). Subsequent studies in experimentally infected animals shed light on the mechanisms by which *T. cruzi* induces microvasculopathy (Andrade et al., 1994; Tanowitz et al., 1999). Initial observations ascribed the formation of vasospasm to the pathogenic activity of endothelins (ETs), a potent class of vasoconstrictor polypeptides (Tanowitz et al., 1999). Of further interest, these workers reported that endothelin-1 (ET-1) expression is up-regulated in parasitized cardiovascular cells (Petkova et al., 2000). Follow-up studies in chronically infected mice demonstrated that cardiac remodeling significantly ameliorated in transgenic lines in which the ET gene was specifically removed from cardiomyocytes, while ablation of this gene in endothelial cells has not significantly reduced heart fibrosis (Tanowitz et al., 2005). Of further interest, the plasma levels of ETs are elevated both in chagasic patients and infected mice (Petkova et al., 2000; Salomone et al., 2001).

While the research linking infection-associated vasculopathy to the function of ETs progressed, our group reported that trypomastigotes generate proinflammatory kinin peptides extravascularly. Follow-up studies indicated that the local activation of the kallikrein-kinin system (KKS) translates into mutual benefits to the host/parasite relationship during the course of chagasic infection (Scharfstein and Andrade, 2011; Scharfstein and Svensjö, 2012). In the present article, we will review the results of these studies and advance the proposition that *T. cruzi* may take advantage of interstitial edema in the inflamed heart to potentiate their infectivity via cooperative signaling of multiple G protein-coupled receptors (GPCRs). The rationale of this hypothesis lies on two fundamental premises: (i) due to the low-grade parasitism observed in chronic infection, there are intermittent "flares" of plasma leakage in the inflamed myocardium (ii) the microvascular edema is temporally linked to the release of parasites from ruptured pseudocysts (Scharfstein and Morrot, 1999).

Studies in various experimental models indicated that tissue culture-derived trypomastigotes (Dm28c) swiftly activate microvascular beds through the activation of the KKS (Todorov et al., 2003; Monteiro et al., 2006; Schmitz et al., 2009; Scharfstein and Andrade, 2011; Andrade et al., 2012). Based on these initial observations, we predicted that the sudden diffusion of plasma-borne constituents (antibodies, complement components, kininogens, ETs) through parasite-laden tissues may affect the delicate host/parasite balance established in the chronically infected myocardium. Although the flagellated trypomastigotes released from pseudocysts may rapidly move away from the primary foci of infection, hence seeking for safer targets elsewhere in the myocardium, we proposed that the transient rise of plasma proteins in the edematous interstitial spaces might favor generation of infection-promoting peptides, such as bradykinin (BK), in the peripheral tissues (Scharfstein et al., 2000; Todorov et al., 2003; Andrade et al., 2012). Before outlining the arguments supporting this working hypothesis, we will present readers with an overview of the essential structural and functional features of the KKS.

## MOLECULAR BASIS OF KKS ACTIVATION AND REGULATION

**FIGURE 1:**
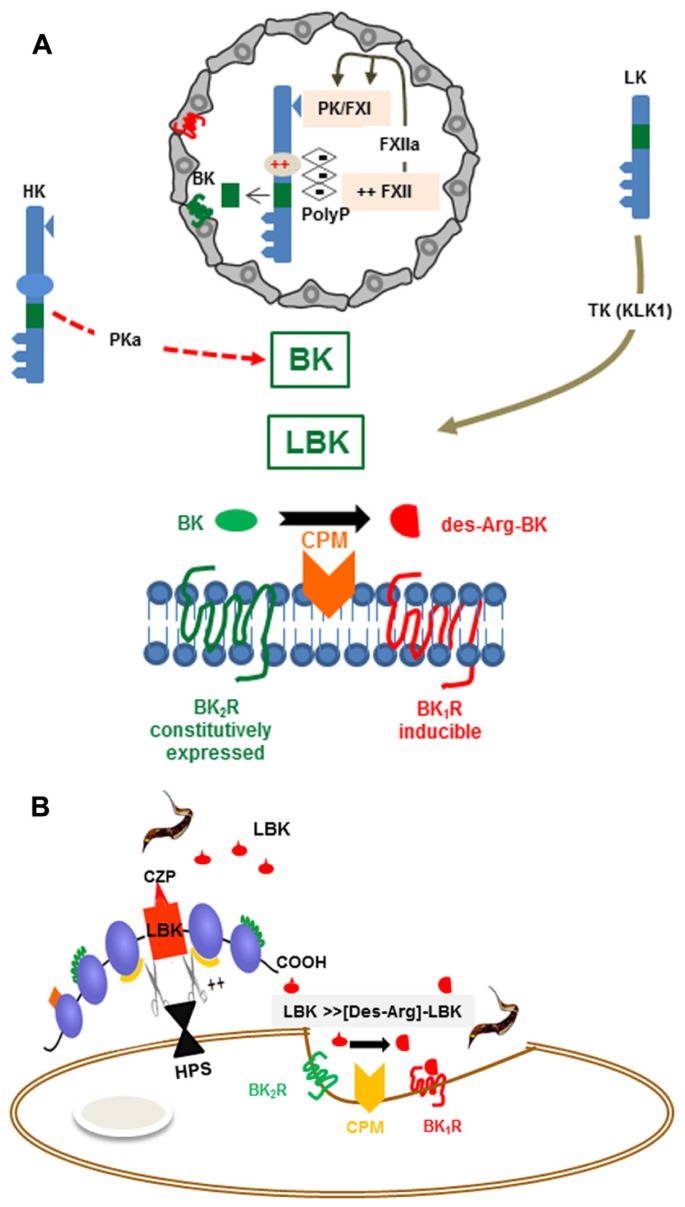
**(A)** Activation of the kallikrein-kinin system *Top*: The scheme depicts platelet-derived polyphosphates (PolyP) acting (intravascularly) as endogenous activators of the KKS (contact phase of coagulation). The proteolytic cascade is initiated as the negatively charged PolyP (long chains) bind to (i) FXII and (ii) HK, via the histidine-rich (D5) domain. PolyP-induced conformational changes in FXII lead to its autocatalytic cleavage. FXIIa then cleaves the plasma kallikrein (PK) zymogen, generating PKa. After several cycles of reciprocal activation between PKa/FXIIa, FXIIa activates FXI, the downstream effector of the intrinsic coagulation pathway, whereas PKa cleaves HK, releasing the internal nonapeptide bradykinin (BK, green rectangle). In inflammatory conditions, the plasma-borne HK/LK diffuse through post-capillary venules. As the extravascular levels of kininogens rise, these kinin-precursor molecules undergo cleavage by tissue kallikrein (TK), which liberates the decapeptide lysyl-BK. Acting by the paracrine mode, the intact form of kinins (BK or Lysyl-BK) binds at high-affinity to BK_2_R, a subtype of BKR that is constitutively expressed in a broad range of cell types. *Bottom*: The scheme depicts the activation requirements of BK_1_R, a GPCR subtype whose expression is strongly upregulated in inflamed/damaged tissues. Generation of high-affinity ligands for the inducible BK_1_R depends on metabolic processing of the intact kinins (BK or lysyl-BK) by the metallopeptidase kininase I (carboxypeptidase N or membrane-bound carboxypeptidase M). **(B)** BK1R is an inducible gateway for *T. cruzi* entry in cardiovascular cells. *T. cruzi* relies on the enzymatic versatility of the major lysosomal cysteine protease (cruzipain) to release LBK from HK molecules docked to the host cell surfaces via binding to heparan sulfate proteoglycans. Once released, the short-lived lysyl-BK is rapidly processed by membrane-bound carboxypeptidase M, generating [des-Arg]-lysyl-BK, which then enhances parasite infectivity through the signaling of the inducible BK_1_R.

Recently implicated in thrombo-inflammatory processes (Müller et al., 2009; von Brühl et al., 2012), the KKS is a hub-like network of proteolytic enzymes which, among other biological functions, release the proinflammatory “kinin” peptides from an internal segment of their plasma-borne precursors, the kininogens. Generation of kinins may involve multiple processing enzymes: in the bloodstream, plasma kallikrein (PK) releases the nonapeptide BK from high molecular weight kininogen (HK) upon activation of the contact system by negatively charged surfaces, such as platelet-derived polyphosphates (**Figure 1A**). In the extravascular spaces, lysyl-BK (LBK) is excised from low molecular weight kininogen (LK) or HK by tissue kallikrein, a serine protease that is constitutively expressed in multiple tissues. It is also known that kinins can be generated by alternative proteases. For example, in chronic inflammation kininogens may be processed by the concerted action of neutrophil elastase and mast cell tryptase, leading to the release of a slightly larger kinin, Met-LBK (Kozik et al., 1998). In the context of infections, kinins can be directly liberated from the kininogens by the action of microbial cysteine proteases, such as gingipain from *Porphyromonas gingivalis* (Imamura et al., 1994), staphopain A from *Staphylococcus aureus* (Imamura et al., 2005), streptopain from *Streptococcus pyogenes* (Herwald et al., 1996), and cruzipain (Del Nery et al., 1997; Scharfstein et al., 2000; Monteiro et al., 2006).

The biological responses mediated by BK and LBK are mediated by bradykinin B2 receptor (BK_2_R), a subtype of heterotrimeric G-protein-coupled BKR (**Figure 1A**) which is constitutively expressed by several cell types, including cardiomyocytes, pain-sensitive neurons, vascular endothelial cells, smooth muscle cells (Leeb-Lundberg et al., 2005). As explained later on in this text, kinins (BK/LBK) also activate BK_2_R expressed by sentinel cells of the innate immune system, such as conventional dendritic cells (DCs; Aliberti et al., 2003; Bertram et al., 2007; Kaman et al., 2009).

Once released from HK/LK, the intact kinins (BK/LBK) have a half-life of <15 s in the plasma, hence must swiftly activate BK_2_R via the paracrine mode (**Figure 1A**). Since excessive activation of the kinin system may have adverse effects to the vascular system, the kinin/BK_2_R pathway is fined-tunned by overlapping regulatory mechanisms, such as (i) down-regulation of surface BK_2_R (ii) degradation of intact kinins by various metallopeptidases, including angiotensin converting enzyme (ACE, kininase II) – a transmembrane di-peptidyl carboxypeptidase (Skidgel and Erdos, 2004) highly expressed in the endothelium lining and, to a less extent, in other cell types, including innate sentinel cells such as monocytes and DCs (Danilov et al., 2003). Besides degrading intact kinins, thus reducing hypotension, ACE increases blood pressure through the formation of angiotensin II, a potent vasopressor octapeptide. Noteworthy, the transmembrane (somatic) enzyme undergoes cleavage by disintegrin and metalloproteinase (ADAM)-type “sheddase” (Parkin et al., 2004), leading to the accumulation of soluble forms of ACE in the blood and other body fluids.

In contrast to the constitutive BK_2_R, the expression of BK_1_R is strongly – but transiently – up-regulated during inflammation (Marceau and Bachvarov, 1998; **Figure 1A**). For example, ET-1 and angiotensin II induce BK_1_R expression through the signaling of ET_A_R and AT_1_ (angiotensin 1 receptor) respectively, leading to activation of phosphoinositide 3-kinase (PI3K) and mitogen-activated protein kinases (MAPK) cascade (Medeiros et al., 2004). BK_1_R is also induced by IL-1β, TNF-α, and IFN-γ via the NF-κB transcription factor (Marceau and Bachvarov, 1998; Medeiros et al., 2004; Moreau et al., 2007). Differently from BK_2_R, the inducible BK_1_R is not triggered by “intact” kinins (BK/LBK). Instead, BK_1_R is activated by the kinin metabolites [des-Arg]-BK or [des-Arg]-LBK, both of which are generated by carboxypeptidase N (CPN)/carboxypeptidase M (CPM) (kininase I)-mediated cleavage of the C-terminal arginine residue of BK/LBK (**Figure 1A**).

## BIOLOGICAL FUNCTIONS OF BKRs

In the cardiovascular system, kinins/BK_2_R control the blood flow through nitric oxide (NO)-dependent vasodilation. In contrast to the beneficial role of kinins in cardiovascular homeostasis, there is evidence that dysregulated BK_1_R signaling drives myocardial fibrosis and impairs heart function in different experimental models (Spillmann and Tschöpe, 2012).

Beyond inducing detrimental cardiac responses, it is well-established that BK_1_R plays a major role in hyperalgesia (Calixto et al., 2004; Gabra et al., 2005; Cunha et al., 2007). Studies in mice subjected to traumatic brain injury revealed that blood-brain leakage and recruitment of inflammatory leukocytes to the CNS is blocked by a specific BK_1_R antagonist (Raslan et al., 2010). Although inflammation is usually initiated through the signaling of the constitutive BK_2_R, the sustenance of the inflammatory response depends on the signaling of endothelial BK_1_R. McLean et al. (2000) were the first to report that the trans-endothelial leukocyte migration is enhanced as result of up-regulated expression/signaling of endothelial BK_1_R. Recent progress in studies of experimental autoimmune encephalitis (EAE) demonstrated that BK_1_R increases the recruitment of pathogenic effector T cells into the CNS (Dutra et al., 2011; Göbel et al., 2011; **Table 1**).

**Table 1:** Role of BKRs in immunity: literature update.

Experimental System	Bradykinin receptor subtype	Functional Roles of BKRs	Reference
*T. cruzi* infection (mice)	BK_2_R	Induction of CD11c+ DC (splenic) maturation and upregulation of effector (type 1) CD4 and CD8 T cells	Monteiro et al. (2006); Monteiro et al. (2007)
*L. donovani* and *L. chagasi* (hamsters and mice)	BK_2_R	Inflammatory edema induced by promastigotes; stimulation of promastigote uptake and modulation of the intracellular growth of leishmania in macrophages	Svensjö et al. (2006)
Visceral Leishmaniasis (mice)	BK_2_R	Immune resistance to acute infection	Nico et al. (2012)
*L. monocytogenes* infection (mice)	BK_2_R	Modulation of innate immunity and infection control	Kaman et al. (2009)
*P. gingivalis* infection-bucal (mice)	BK_2_R	Upregulation of Th1 and Th17 responses (submandibular lymph node)	Monteiro et al. (2009)
*In vivo* leukocyte trafficking across mesenteric postcapillary venules (mice)	BK_1_R	Trans-endothelial leukocyte migration	McLean et al. (2000)
EAE (mice)	BK_1_R	Th1 and Th17 responses development and clinical progression of EAE	Dutra et al. (2011)
		Induction of blood brain barrier disruption and T cell transmigration into the CNS	Göbel et al. (2011)
		Limiting infiltration of pathogenic subsets of effector CD4 T cells into the CNS	Schulze-Topphoff et al. (2009)
EAE (mice)	BK_1_R	Th1 and Th17 responses development and clinical progression of EAE	Dutra et al. (2011)
		Induction of blood brain barrier disruption and T cell transmigration into the CNS	Göbel et al. (2011)
		Limiting infiltration of pathogenic subsets of effector CD4 T cells into the CNS	Schulze-Topphoff et al. (2009)
*In vitro* DC migration (human)	BK_2_R	Stimulation of DC migration	Bertram et al. (2007)
	BK_1_R	Inhibition of DC migration	Gulliver et al. (2011)
Allergic inflammation (mice)	BK_1_R/BK_2_R	Induction/inhibition of migration and activation of eosinophils	Vasquez-Pinto et al. (2010)
	BK_2_R	Induction of DC maturation and IL-12-dependent Th1 polarization	Aliberti et al. (2003)

More recently, a growing number of studies indicated that immune resistance against infection by parasitic protozoan (Monteiro et al., 2006, 2007; Svensjö et al., 2006; Nico et al., 2012; Scharfstein and Svensjö, 2012) and bacterial pathogens (Kaman et al., 2009) is critically dependent on activation of innate sentinel cells via the BK_2_R pathway (**Table 1**).

## BKRs: DOMAIN COMPARTMENTALIZATION, REGULATION, AND SIGNAL TRANSDUCTION PATHWAYS

Stably expressed at the cell surface, the constitutively expressed BK_2_R is rapidly desensitized/internalized via GRK4α-mediated receptor phosphorylation upon ligand binding, thus yielding a transient signaling response (Blaukat et al., 2001). Using BK_2_R-fusion constructs, Enquist et al. (2007) revealed that upon BK binding, the internalized receptors colocalize with transferring in endosomes, prior to entry in the arrestin-dependent, clathrin-mediated recycling pathway. A second trafficking pathway, described in smooth muscle cells and fibroblasts, indicated that BK also promotes the redistribution of BK_2_R and their coupling G proteins to caveolar rafts (de Weerd and Leeb-Lundberg, 1997).

Although the cellular systems vary from one report to another, it is well-established that BK binding to BK_2_R promotes the formation of BK_2_R homodimers and oligomer complexes of higher order (Leeb-Lundberg et al., 2005). Kang et al. (2004) reported that BK_2_R and BK_1_R form heterodimers in transfected HEK293 cells. Notably, BK_2_R also forms heterodimeric complexes with-type-1 angiotensin receptors (AT_1_) in vascular smooth muscle cells and transfected HEK293 (AbdAlla et al., 2001).

As to the signal transduction pathways, it is well established that BK frequently induces BK_2_R-dependent [Ca^2+^]_i_ transients and PLC-β-dependent hydrolysis of phosphoinositides, both of which commonly coupled to the activation of PKC isoenzymes α, ε, and ξ. Although BK_2_R often signals through Gα_q_ and Gα_i_, in some cellular systems BK-driven activation of the constitutively expressed BKR is coupled to Gαs and Gα12/13 (Leeb-Lundberg et al., 2005). Similar to the effects induced by other GPCR agonists, BK sequesters BK_2_R along with Gα_q_ and Gα_i_ in caveolar compartments of smooth muscle cells (de Weerd and Leeb-Lundberg, 1997).

In fibroblasts, a cell-type often used in models studies of *T. cruzi* interaction with non-professional phagocytic cells, BK_2_R activation is associated with transient tyrosine phosphorylation and activation of focal adhesion kinase as well as other focal-adhesion substrates (Leeb-Lundberg et al., 1994). In another study with fibroblasts, BK stimulated peripheral actin microspikes and membrane ruffling via activation of Cdc42 and Rac-1 (Kozma et al., 1995). Studies with endothelial cells showed that BK, acting as a potent vasodilator, transiently stimulates endothelial cell production of NO via endothelial nitric oxide synthase (eNOS) through mechanisms involving both calcium-dependent and phosphorylation cascades mediated by PI3K/Akt (Kuhr et al., 2010).

## KKS HAS A DUAL ROLE IN EXPERIMENTAL CARDIOMYOPATHIES

All the components of the KKS are present in the heart. In addition to contributing to vascular tone and inflammation in the cardiac tissues, the KKS influences extent of extracellular matrix (ECM) remodeling, angiogenesis, and stem cell mobilization (Spillmann et al., 2006). Of possible relevance to the understanding of KKS function in CCM, experimental studies in mouse models of diabetic cardiomyopathy and myocardial ischemia indicate that specific alterations in kinin homeostasis may either ameliorate or worsen cardiac pathology after cardiac pathology (Spillmann and Tschöpe, 2012).

As mentioned earlier, the beneficial effects of ACE inhibitors are due to their dual biological functions: these potent drugs block ACE-driven conversion of angiotensin I into the vasopressor angiotensin II and inhibit the enzymatic breakdown of kinins (BK_2_R agonists), thus increasing the half-life of “intact” kinins in the circulation. Noteworthy, ACE inhibitors reduce myocardial damage (e.g., reduction of infarction size) yet these effects were nullified upon treatment with BK_2_R antagonists or in BK_2_R-deficient mice (Yang et al., 2001), thus indicating that signaling of BK_2_R has a major function in the remarkable protective effects of ACE inhibitors.

Studies in streptozotocin (STZ)-induced models of diabetic cardiomyopathy have shown that early left ventricle (LV) and systolic dysfunction is improved in mice overexpressing tissue kallikrein (KLK1), whereas wild-type heart develop massive fibrosis (interstitial and perivascular) due to focal accumulation of collagen and fibrillar fragmentation. Although KLK1 has a pro-collagenase activity by itself and may activate matrix metalloproteinases (MMPs), the host-protective effects of KLK1 overexpression in diabetic cardiomyopathy were abolished upon treatment with HOE-140 (BK_2_R antagonist), thus implying that KLK1-mediated release of kinins improves cardiac function. Additional studies suggested that activation of the KLK1/BK_2_R pathway reduces cardiac fibrosis through the activation of the plasminogen activator/MMP2-dependent fibrinolytic cascade (Spillmann et al., 2006). Additional studies revealed that BK_2_R signaling that BK_2_R signaling improves cardiac function by (i) reducing apoptosis and chamber dilatation in the myocardium (Chao et al., 2008), (ii) optimizing angiotensin II-induced (NO-dependent) neovascularization (Munk et al., 2007), (iii) restoring S2a-mediated sarcoplasmatic Ca^2+^ uptake (Tschöpe et al., 2005), and/or (iv) promoting homing of endothelial progenitor cells to ischemic muscles (Kränkel et al., 2008).

## *T. cruzi* TRYPOMASTIGOTES LIBERATE KININS FROM SURFACE-BOUND KININOGENS VIA CRUZIPAIN

The first clues suggesting that *T. cruzi* is equipped with a kininogenase came from enzymatic analysis of the substrate specificity of cruzipain (Del Nery et al., 1997), a lysosomal-like cysteine protease classified as member of clan A of the C1 peptidase family (Cazzulo et al., 2001; Alvarez et al., 2012). At first sight, the discovery that cruzipain acted as a “kininogenase” seemed paradoxical because kininogens are members of the cystatin family of cysteine protease inhibitors, hence rely on cystatin-like domains to potently inactivate papain-like enzymes, including cruzipain itself (Stoka et al., 1995). Consistent with this, *in vitro* data showed that cruzipain hydrolyzes soluble forms of HK at slower rates as compared to tissue kallikrein. This conundrum was settled by awareness that HK binds to negatively charged sulfated proteoglycans, such as heparan or chondroitin sulfates via the histidine-rich positively charged motif (D5_H_) localized at the C-terminal end of the BK (D4) sequence (Renné et al., 2000; Renné and Muller-Esterl, 2001). Based on this information, Lima et al. (2002) hypothesized that the spatial orientation of cell-bound HK docked to heparan sulfate proteoglycans was not suitable for cruzipain binding and inactivation by the cystatin-like inhibitory domain (**Figure 1B**). Indeed, model studies performed with cruzipain and HK in the test tube offered circumstantial support to this hypothesis (Lima et al., 2002): the addition of heparan sulfate (at optimal concentrations) drastically reduced the cysteine inhibitory activity of soluble HK on cruzipain while reciprocally increasing the catalytic efficiency of the parasite protease, measured with synthetic peptides flanking the BK sites. Consistent with these results, heparan sulfate potentiated HK processing by cruzipain, generating multiple HK breakdown products and promoting accelerated kinin release (**Figure 1B**). Combined, these biochemical studies suggested that the substrate specificity of the parasite protease was re-directed as result of reciprocal interactions between sulfated proteoglycans with the substrate (HK) and protease (cruzipain) molecules (Lima et al., 2002), hence increasing the efficiency of the kinin-release reaction in peripheral sites of infection.

Previously characterized as a therapeutic target of Chagas disease (McKerrow et al., 2009), cruzipain is a remarkably versatile virulence factor of *T. cruzi*. Beyond activation of the KKS (see below), the proteolytic activity of cruzipain was implicated in mechanisms of parasite virulence/pathogenicity, such as lence/pathogenicity, such as (i) enhancement of trypomastigote invasion and intracellular replication of amastigotes in cardiomyocytes (Meirelles et al., 1992; Scharfstein et al., 2000), (ii) degradation of proinflammatory chemokines (Benítez-Hernández et al., 2010), and (iii) subversion of innate microbicidal responses in parasitized macrophages through interference with the activation of NF-κB (Doyle et al., 2011).

## KININ RELEASE IN PERIPHERAL SITES OF *T. cruzi* INFECTION DEPENDS ON THE COOPERATIVE ROLES OF tGPI AND CRUZIPAIN

Vertebrate hosts may control infection by rapidly mobilizing soluble and cellular-based microbicidal systems that destroy pathogen invaders at the cost of limited self-tissue destruction. Perturbations of steady-stated tissue homeostasis are sensed by sentinel cells of the innate immune system through specialized pattern-recognition receptors (PRRs; Medzhitov and Janeway, 1997). In many infections, the activation of PRRs lead to rapid secretion of pre-formed vasoactive mediators by innate sentinel cells (e.g., eicosanoids, leukotrienes, chemokines, TNF-α), which then activate the endothelium lining, rendering them sticky for circulating neutrophils. Further downstream, vasoactive mediators generated/released at the neutrophil/endothelial interface impair the integrity of the endothelial barrier, hence opening the “flood gates” (DiStasi and Ley, 2009).

**FIGURE 2:**
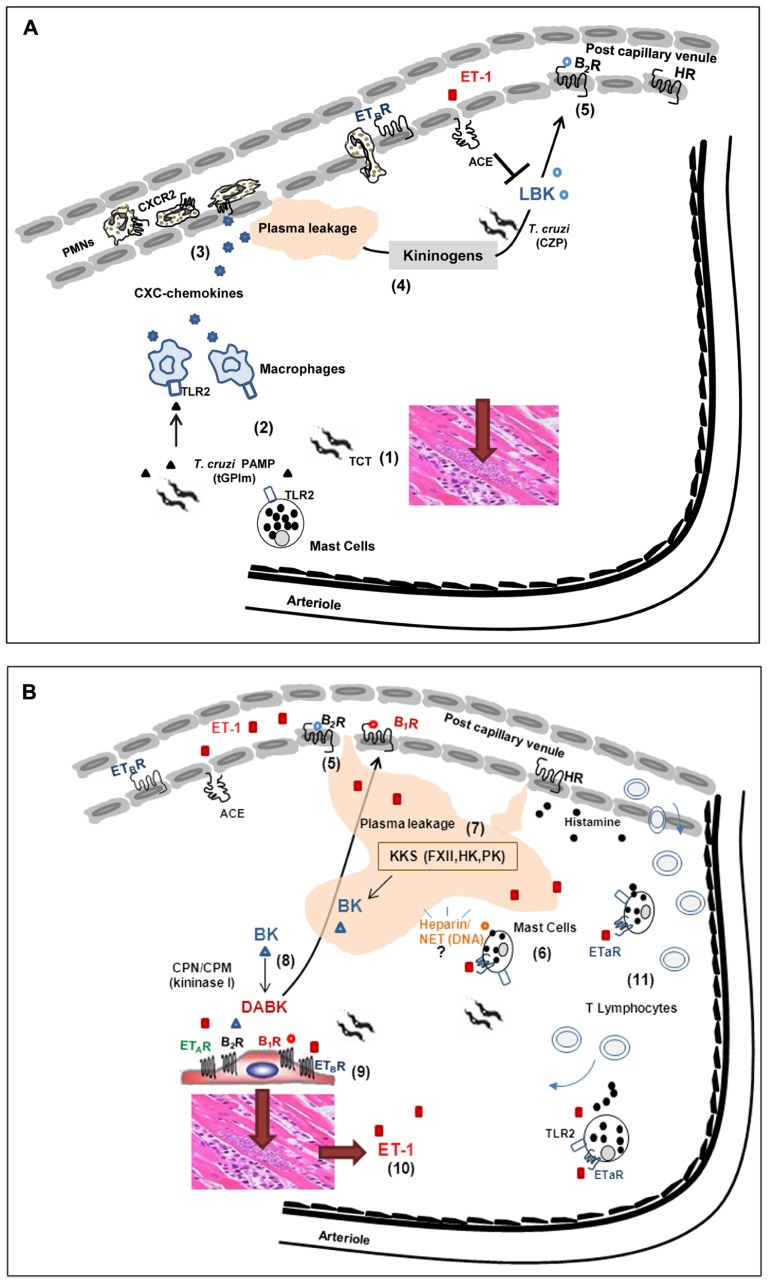
**Influence of interstitial edema on host/parasite balance in the myocardium: lessons from studies with the Dm28c strain of *T. cruzi***. **(A)** (1) Despite the scanty parasitism in the myocardium of chronic chagasic patients, parasite-containing pseudocysts occasionally burst, releasing high numbers of pro-inflammatory trypomastigotes into the surrounding interstitial spaces. (2) Innate sentinel cells, such as macrophages and/or mast cells, initiate inflammation through the sensing of tGPI, a TLR2 ligands shed by trypomastigotes. (3) In response to TLR2 engagement, tissue macrophages release CXC chemokines, which in turn activate neutrophils/endothelium via CXCR2. (4) Vascular permeability is increased, leading to the diffusion/accumulation of plasma proteins (including kininogens) through parasite-laden interstitial spaces. Once bound to heparan sulfate proteoglycans, HK suffers proteolytic attack by cruzipain (CZP) secreted by trypomastigotes. (5) The released kinins (LBK) amplify plasma leakage through the activation of endothelium BK_2_R. The levels of intact kinins keep rising, despite the counter-regulatory role of ACE. **(B)** (6) Whether originating from plasma (following diffusion to sites of infection) or secreted by parasitized cardiomyocytes, ET-1 activates perivascular mast cells via ETAR. Upon degranulation, the mast cell release histamine (which further increases plasma leakage) and heparin, in the peripheral tissues. (7) The plasma “contact” phase system may be activated by mast cell-derived heparin and/or other negatively charged molecules of endogenous origin, such as DNA present in extracellular traps. Once activated by FXIIa, PKa releases BK from HK, thus propagating the proteolytic cascade. (8) Intact kinins are metabolized by kininase I (CPM and/or CPN), generating [des-Arg]-BK, which in turn activate BK_1_R (whose expression is up-regulated in a broad range of heart cells, including cardiomyocytes-shown at the center of the illustration). (9) The parasites take advantage of the local availability of infection-promoting peptides (ET1, BK and/or [des-Arg]-kinins) to persistently invade cardiovascular cells through the signaling (“cross-talk”) between BK_2_R/BK_1_R/ET_A_R/ET_B_R. (10) Intracellular parasite outgrowth leads to increased expression of pro-oxidative/pro-fibrotic ET-1 and proinflammatory chemokines. (11) Adverse cardiac remodeling ensues, as result of vicious cycles of ET-1-driven triggering of cardiac mast cells, endothelial activation and BK1R-driven recruitment of pathogenic subsets of effector CD8^+^ T cells into the heart.

Studies in a mouse model of subcutaneous (footpad) infection provided the first evidence that Dm28c TCTs (tissue culture trypomastigotes) induce inflammatory edema through the activation of the kinin system (Todorov et al., 2003; **Figure 2A**). A footpad edema potentiated by the ACE inhibitor captopril was observed 2 h p.i. in wild-type B6 mice, but not in BK_2_R-deficient mutants. Using pharmacological tools, we showed that the constitutively expressed BK_2_R orchestrated the early phase (2 h) edema, whereas BK_1_R (acting in “cross-talk” with BK_2_R) sustained the inflammatory response (24 h; Todorov et al., 2003). Intriguingly, Dm28c epimastigotes failed to induce a conspicuous inflammatory edema via the kinin pathway despite the fact that these vector-borne non-infective forms express abundant levels of cruzipain. In a subsequent study, it became clear, for reasons that will be explained further below, that cruzipain was necessary but not sufficient to generate kinins in hamster cheek pouch (HCP) topically exposed to Dm28c epimastigotes.

Insight of the functional interplay between Toll-like receptor (TLR)-driven innate responses and the KKS emerged from analysis of the mechanisms underlying TCT-evoked microvascular leakage responses induced by topically applying the pathogen to the HCP (Monteiro et al., 2006). This unconventional system proved advantageous because it dispensed the use of needle to inject parasites, hence avoiding activation of the KKS by bleeding and traumatic injury. Using intravital microscopy, we found that Dm28c TCTs induced discrete plasma leakage responses in the HCP within a few minutes of parasite application. Noteworthy, the microvascular reactions were attenuated by HOE-140 (BK_2_R antagonist) or by pretreating the TCTs with an irreversible cruzipain inhibitor *N*-methyl-piperazine-Phe-homoPhe-vinyl sulfone (K11777), an anti-parasite drug that will be soon tested in clinical trials (Engel et al., 1998; McKerrow et al., 2009).

Monteiro et al. (2006) highlighted the fact that purified cruzipain (activated) topically applied to the HCP failed to induce significant plasma leakage even in the presence of ACE inhibitors. However, purified (activated) cruzipain induced a strong BK_2_R-driven leakage response when it was applied in combination to purified HK. The dependence on a supply of exogenous HK suggested that, in resting state conditions (i.e., in the absence of inflammation), the levels of endogenous kinin-precursor proteins in the interstitial spaces are insufficient to allow for significant activation of the kinin system. Based on these observations, Monteiro et al. (2006) predicted that Dm28c TCTs might bear developmentally regulated proinflammatory molecules (i.e., absent in Dm28c epimastigotes) which control the rate-limiting step of KKS activation in parasite-laden tissues: the influx of the plasma-borne kininogen (cruzipain substrate) into peripheral sites of *T. cruzi* infection (**Figure 2A**). Seeking to identify these factors, Monteiro et al. (2006) then turned their attention to tGPI, a mucin-linked lipid anchor previously characterized by Almeida and Gazzinelli (2001) as a TLR2 ligand that was expressed at high-levels exclusively in TCTs. Consistent with the working hypothesis, Dm28 TCTs failed to evoke inflammatory edema in TLR2^-/-^ mice or in BK_2_R^-/-^ mice (Monteiro et al., 2006). In contrast, these infective parasites evoked a prominent edema both in wild-type mice and TLR4 mutant (C3H/HeJ). These results argued against a role for GIPL, a lipid anchor of epimastigotes previously characterized as TLR4 ligand (Oliveira et al., 2004), in the activation pathway that generates vasoactive kinins in peripheral sites of infection.

Next, Monteiro et al. (2006) studied the functional interplay between TLR2 and the KKS by injecting TLR2-deficient mice with a parasite suspension supplemented (or not) with purified HK. Strikingly, the injection of HK in infected TLR2^-/-^ mice rescued the edema response. Moreover, the edema responses which HK induced in TLR2^-/-^ infected mice were abolished by HOE-140, or alternatively, by pre-incubating the TCTs with the irreversible cruzipain inhibitor K11777. Combined, these results supported the hypothesis that TCTs induce the initial leakage/accumulation of the HK (cruzipain substrate) in peripheral tissues via TLR2, whereas cruzipain amplifies this inflammatory response through the release of BK_2_R agonist, at the downstream end of the inflammatory cascade (**Figure 2A**).

In order to further confirm this hypothesis, Monteiro et al. (2006) injected (s.c.) purified tGPI, combined or not to activated cruzipain, in wild-type naïve mice or in TLR2^-/-^ or BK_2_R^-/-^ mutants. Edema measurements showed that tGPI/cruzipain potently induced microvascular responses via the TLR2/BK_2_R-pathway and these proinflammatory effects were further potentiated by ACE inhibitors. Beyond their effects on the microcirculation, the released kinins link TLR2-driven inflammation to innate immunity by triggering BK_2_R expressed on resident/migrating DCs, converting them into T_H_1-directing antigen-presenting cells (APCs; Monteiro et al., 2006). Conversely, ACE counter-modulates T_H_1-polarization via the trans-cellular the TLR2/BK_2_R pathway by degrading BK/LBK, an endogenous signal that drives DC maturation (Aliberti et al., 2003; Monteiro et al., 2006, 2007; Scharfstein et al., 2007).

## NEUTROPHIL LINKS TLR2/CXCR2 TO THE PROTEOLYTIC (KKS) PHASE OF INFLAMMATION

A key event at early stage of infection, the interaction of circulating neutrophils with the endothelium has profound effects on the outcome of the inflammatory process. One of the most common consequences of the interactions that take place on the luminal side of post-capillary vessels is the increased vascular permeability, a biological response that leads to the accumulation of protein-rich edema fluid in interstitial spaces. Although the list of endogenous soluble factors that increase vascular permeability is extensive, they usually impair the integrity of the endothelial barrier through the triggering of [Ca^2+^]_I_ – the via signaling of heterotrimeric G-proteins, i.e., a pathway required to induce myosin-dependent contraction and junctional disruption of the endothelial cells.

In the HCP studies described in the previous section, Monteiro et al. (2006) observed that circulating leukocytes rapidly adhered to the luminal face of post-capillary venules. Complementary studies performed in neutrophil-depleted wild-type mice revealed that Dm28 TCTs failed to evoke a conspicuous edema in these animals. Similar to the pharmacological maneuvers employed with TLR2^-/-^ mice, already described, the deficient phenotype of neutrophil-depleted mice was rescued upon injection of TCT supplemented with HK (Monteiro et al., 2006). Collectively, these results supported the concept that neutrophils, acting at the early stages of the inflammatory response, link innate immunity (TLR2-driven) to the KKS by driving the diffusion/accumulation of plasma-borne kininogens into parasite-laden tissues (**Figure 2A**).

In a subsequent study, Schmitz et al. (2009) investigated whether macrophages could also play a role in the trans-cellular “cross-talk” mediated by TLR2/BK_2_R. First, they asked whether macrophages incubated with TCTs or tGPI *in vitro* stimulated the secretion of CXC chemokines (KC/MIP-2). The results revealed that wild-type macrophages robustly responded to Dm28c TCTs (but not to epimastigotes), whereas TLR2^-/-^ macrophages were unresponsive to both stimuli. Turning to the HCP model, intravital microscopy studies showed that the drug repertaxin (CXCR2 antagonist) blocked leukocyte accumulation in the microvascular beds of HCP topically exposed to TCTs and inhibited the paw edema in *T. cruzi*-infected mice (Schmitz et al., 2009). Collectively, these findings supported the concept that Dm28c TCTs evoke inflammatory edema via the TLR2/CXCR2/BK_2_R axis, a sequential pathway of activation forged by the trans-cellular “cross-talk” of tissue resident macrophages, neutrophils and the endothelium (**Figure 2A**).

## FURTHER EXPANSION OF THE INFLAMMATORY WAVE: THE CONVERGENCE BETWEEN ENDOTHELIN AND KININ PATHWAYS

Encoded by distinct genes, ET-1, 2, and 3 are closely related peptides expressed by endothelial cells, cardiac myocytes, and cardiac fibroblasts (Goto, 2001; Kedzierski and Yanagisawa, 2001). Synthesized as pre-pro-endothelin, these precursor proteins are cleaved by ET-converting enzymes (ECE) forming big-endothelin, which upon further processing yields 21 amino acids ET peptides that activate cardiovascular cells via GPCRs subtypes, ET_A_R and ET_B_R (Dhaun et al., 2007).

Apart from its powerful vasoconstrictor effects, the pleiotropic ET-1 induces plasma exudation (Filep et al., 1993; Sampaio et al., 2000). Hemodynamic changes such as those provoked by shear stress are sensed by the endothelium, which responds by activating eNOS and early up-regulating ET-1 mRNA. Once released, NO and ET-1 modulate tissue homeostasis extravascularly through the activation of other cell types, such as mast cells (Maurer et al., 2004).

Recently, Andrade et al. (2012) investigated the functional interplay between the kinin and ETs pathways at the early stages of infection by Dm28c TCTs. Remarkably, the application of subtype-specific antagonists of ET_A_R or ET_B_R, or the BK_2_R antagonist (HOE-140) on the HCP prior to TCTs markedly reduced (~70%) leukocyte accumulation in microvascular beds. In addition, these endothelin receptor (ETR) antagonists blunted plasma leakage in the hamster cheek pouch and blocked the inflammatory edema in *T. cruzi*-infected mice. Collectively, these results indicated that ETRs (both subtypes) and BKRs, may propagate the inflammatory wave intiated via TLR2/CXCR2 through the proteolytic activation of the KKS.

## MAST CELLS MAY COUPLE TLR2 TO ETRs/BKRs-DRIVEN PATHWAYS OF INFLAMMATION

Levick et al. (2010) have recently drawn attention to the importance of cardiac mast cells in adverse myocardial remodeling. Given the precedent that mast cells sense *Mycobacterium tuberculosis* via TLR2 (Carlos et al., 2009), Andrade et al. (2012) have speculated that Dm28c TCTs may directly activate ET-positive mast cells (Ehrenreich et al., 1992) via TLR2, perhaps upregulating the extravascular levels of ETs in the chagasic myocardium. Although plausible, the pathogenic role of TLR2-positive cardiac mast cells in CCM was not object of systematic investigations so far. Focusing on other aspects of chagasic pathology, Meuser-Batista et al. (2011) have recently reported that cardiac mast cells die by apoptosis in CBA-infected mice, presumably reflecting reduced production of stem cell factor in chagasic heart.

Studies in other disease settings have shown that ET-1 activates ET_A_R-positive mast cells in autocrine manner, inducing secretion of potent vasoactive mediators, such as histamine, leukotriene C4 (Yamamura et al., 1994), and TNF-α (Coulombe et al., 2002). Of further interest, there is evidence that ET-1 induces metalloproteinase-driven ventricular remodeling in models of chronic heart pressure/volume overload (Murray et al., 2004; Janicki et al., 2006) and modulate cardiac contractility through the induction of mast cell degranulation via ET_A_R (Eszlári et al., 2008). Although the role of the ET-1/mast cell axis was not directly investigated in CCM, there is evidence that plasma levels of ET-1 are elevated both in chagasic patients and experimentally infected mice (Petkova et al., 2000; Salomone et al., 2001), presumably reflecting increased shear stress or other infection-associated hemodynamic alterations in these individuals. Furthermore, as highlighted earlier in this text, the expression of ETs is up-regulated in parasitized cardiomyocytes (Tanowitz et al., 2005). Thus, irrespective of the source of ET-1, it is conceivable that the sudden rise in the extravascular levels of this potent pro-oxidative mediator may lead to the activation of perivascular ET_A_R-positive mast cells in the chagasic heart. According to this hypothetical scenario, mast cell degranulation via ET_A_R may release histamine, chemokines, along with a myriad of vasoactive mediators, in the parasite-ladden cardiac tissues (**Figure 2B**). More recently, Nascimento et al. (2012) have started to investigate this hypothesis by interfering with mast cell function in HCP topically exposed to Dm28 TCTs. Using mast cell stabilizers, we found that plasma leakage (BK_2_R-driven) was indeed inhibited, thus suggesting that mast cell degranulation is required for overt activation of the KKS in peripheral sites of chagasic infection (**Figure 2B**).

Thus far, the mechanisms by which mast cells may propagate the KKS cascade in peripheral sites of *T. cruzi* infection remain unknown. In an interesting precedent coming from mouse models of allergic inflammation, Oschatz et al. (2011) have recently proposed that heparin (a mast cell storage product) acts as a typical endogenous “contact” activator, i.e., it activates PKa in FXIIa/HK-dependent manner, thereby releasing BK. Alternatively, parasite-induced activation of cardiac mast cells may activate the KKS through the formation of DNA-containing extracellular traps (von Köckritz-Blickwede et al., 2008). Further studies may determine if the ET/mast cell/KKS pathway plays an important role in the pathogenesis of CCM.

## BK_2_R IS ESSENTIAL FOR DEVELOPMENT OF ACQUIRED IMMUNE RESISTANCE TO CHAGASIC INFECTION

Comprising a heterogeneous population of professional APCs, DCs are widely but sparsely distributed in peripheral tissues and lymphoid organs (Shortman and Naik, 2007). Strategically positioned in T cell-rich areas of secondary lymphoid tissues, the resident DCs are specialized in antigen-presentation to CD4^+^ and CD8^+^ T cells. In steady-state conditions, immature DCs present MHC-restricted antigen peptides to virgin T cells in the absence of co-stimulatory molecules, hence contributing to the maintenance of peripheral tolerance (Sela et al., 2011). However, during infection, the immature DCs sense “danger” motifs expressed by pathogens through distinct families of PRRs, such as TLRs or intracellular NOD2-like receptors (NLR; Akira, 2009). In addition, conventional DCs may sense the threat to tissue integrity via receptors for endogenous proinflammatory mediators, such as ATP, uric acid (Sansonetti, 2006), and BK (Aliberti et al., 2003; Monteiro et al., 2007). Stabilized by cognate interactions with co-stimulatory molecules (CD80/86, CD40, and MHC), the prolonged encounters between antigen-bearing DCs and naïve T cells are essential for TCR activation. During the course of DC/T cell interaction, the “mature” APCs deliver T_H_ polarizing cytokines, such as IL-12p-70, which is critically required for T_H_1 development.

Efforts to characterize the activation pathways controlling DC maturation in the context of *T. cruzi* infection have initially converged to nucleic acid-sensing TLRs (3, 7, and 9). In a key study, Caetano et al. (2011) showed that transgenic mice strains which lack functional UNC93B1 as well as functional endosomal TLRs (TLR3, 7, and 9), were as susceptible to *T. cruzi* infection as mice deficient in TLR3/7/9 (Caetano et al., 2011). In the same study, it was documented that *T. cruzi*-infected macrophages and DCs from 3 day mice displayed low IL-12p40 and INF-γ responses. Based on these results, it was inferred that recognition of intracellular parasites require UNC93B1-driven translocation of the nuclei acid-sensing TLRs from the endoplasmic reticulum to the endolysosomes (Caetano et al., 2011). In spite of this conceptual advance, other studies suggest that DCs might sense *T. cruzi* through TLR-independent pathways. For example, Kayama et al. (2009) showed that *T. cruzi* induces maturation (up-regulation of MHC class II, CD40, and CD86) of MyD88^-/-^TRIF^-/-^ mice bone-marrow-derived DCs as efficiently as wild-type bone-marrow-derived DCs. Using fetal liver DCs as target cells, these authors linked the *T. cruzi*-induced responses (IFN-γ production and DC maturation) to the activation of the NFATc1 pathway. These results implied that innate immunity is not exclusively controlled by nucleic acid-sensing TLRs.

Several years ago, we reported that that BK, acting as a typical endogenous danger signal (i.e., T_H_1-directed endogenous adjuvant) induced DC maturation (IL-12 and up-regulated expression of co-stimulatory molecules) via BK_2_R (Aliberti et al., 2003). In a key finding, we subsequently reported that BK_2_R-deficient mice succumbed to acute challenge by Dm28c TCTs (i.p. route). Analysis of the immune dysfunctions underlying the susceptible phenotype of BK_2_R^-/-^ mice at early stages of infection showed a modest, but significant drop in the frequency of intracardiac type-1 effector T cells. Intriguingly, however, as the acute infection progressed in BK_2_R^-/-^ mice, the immune deficiency was intensified and generalized, involving both the extra-lymphoid and lymphoid compartment. Of note, the decayed T_H_1 response of BK_2_R^-/-^ was accompanied by a corresponding rise in IL-17-producing T cells (T_H_17). The premise that the deficient adaptive response of BK_2_R^-/-^ mice was a secondary manifestation resulting from impaired BK_2_R^-/-^ DC maturation was confirmed by systemically injecting wild-type BK_2_R^+/+^ DCs into the susceptible BK_2_R^-/-^ mice, prior to pathogen injection. Remarkably, this DC transfer maneuver rendered the recipient BK_2_R^-/-^ mice resistant to acute *T. cruzi* challenge, and restored their capability to generate protective IFN-γ-producing CD4^+^ CD44^+^ and CD8^+^ CD44^+^ effector T cells, while conversely suppressing the potentially detrimental T_H_17 (CD4^+^ subset) anti-parasite responses.

In the same study, Monteiro et al. (2007) further demonstrated that Dm28c TCTs potently activated BK_2_R^+/+^ CD11c^+^ DCs (splenic origin) but not BK_2_R^-/-^ DCs, using IL-12 secretion and expression of co-stimulatory molecules (CD86, CD80, CD40) as read-out for DC maturation *in vitro*. Of further interest, K11777-treated trypomastigote failed to robustly activate wild-type DCs, thus linking generation of the BK_2_R agonist (DC maturation signal) to the proteolytic activity of cruzipain. Noteworthy, Dm28c TCTs also induced the maturation of (splenic) TLR2^-/-^ CD11c^+^ DCs and TLR4 mutant (C3H/HeJ) via BK_2_R, thus precluding cooperative signaling between this GPCR and either one of these surface PRRs. Admittedly, these results do not exclude the possibility that enhanced parasite uptake via the BK_2_R pathway might have indirectly facilitated nucleic acid-sensing by TLRs residing in endolysosomes of immature splenic DCs. In any case, whether acting as a classical sensor receptor, and/or as a upstream pathway that potentiates TLR-signaling by parasite DNA or ssRNA, these results are in line with the concept that kinin “danger” signals proteolytically released by TCTs convert splenic BK_2_R^+/+^ DCs into inducers of type 1 immunity (Monteiro et al., 2007). Considering that the splenic parenchyma is continuously exposed to plasma proteins, it is conceivable that flagellated trypomastigotes navigating through the splenic stroma might be faced with abundant levels of blood-borne kininogens, most likely associated to ECM or cell-surface sulfated proteoglycans. Accordingly, we may predict that antigen-bearing CD11c^+^ DCs (bearing *T. cruzi* antigens) residing in the spleen (and/or liver) stroma are converted into T_H_1 inducers following exposure to high-levels of kinin “danger” signals. To this date, studies of BK_2_R function in human DCs were limited to lineages derived from monocytes exposed to granulocyte-macrophage colony-stimulating factor (GM-CSF)/IL-4. Using this experimental model, Bertram et al. (2007) reported that BK_2_R signaling promotes DC migration although BK, *per se*, did not induce the maturation of human DCs. However, in subsequent studies Kaman et al. (2009) suggested that BK_2_R signaling potentiates the maturation of DCs previously “primed” via TLR4. In view of the marked phenotypic heterogeneity of DCs (Shortman and Naik, 2007), studies with a more representative range of human DCs are required to determine whether BK_2_R is a key sensor of *T. cruzi.*

In conclusion, the analysis of BK_2_R function in different models of acute *T. cruzi* infection strongly suggests that activation of the kinin system fuels anti-parasite immunity. In the next section we will review evidences indicating that generation of kinins in sites of infection may also benefit *T. cruzi*, hence translating into mutual benefits to the host–parasite equilibrium.

## TCTs RELY ON CRUZIPAIN ACTIVITY TO INVADE CARDIOVASCULAR CELLS VIA THE BK_2_R PATHWAY

As mentioned earlier, studies of *T. cruzi* interaction with mouse cardiomyocytes showed that membrane-permeable irreversible inhibitors of cruzipain impaired trypomastigote invasion and halted intracellular growth of amastigotes (Meirelles et al., 1992). While the studies on cruzipain-mediated pathways of invasion were in progress, Tardieux et al. (1992) reported that *T. cruzi* invades non-phagocytic cells through the induction of calcium-regulated pathway of lysosomal exocytosis. Subsequent studies revealed that the oligopeptidase B-mediated processing of a polypeptide precursor accumulating in the *T. cruzi* cytoplasm generated the calcium-inducing signal (Caler et al., 1998; Morty et al., 1999) that propelled parasite internalization. In a parallel development, Leite et al. (1998) reported evidences that GPCRs were the signal transducers of the [Ca^2+^]_i_-inducing signals generated by the oligopeptidase B-dependent peptide. Consistent with their working hypothesis, parasites genetically deficient in oligopeptidase B showed impaired infectivity, suggesting that proteolytic generation of the cytoplasmic Ca^2+^-inducing signal was indeed required for the development of infective phenotype. In another interesting study, Santana et al. (1997) described the biochemical properties of Tc80, a serine protease displaying collagenase activity. Subsequently characterized as a prolyl oligopeptidase, Tc80 localizes in a vesicular compartment close to the flagellar pocket of trypomastigotes. Synthetic inhibitors of Tc80 potently inhibited *T. cruzi* (Y strain) invasion of non-phagocytic cells without interfering with the [Ca^2+^]_i_-inducing activity of parasite extracts (Grellier et al., 2001).

**FIGURE 3:**
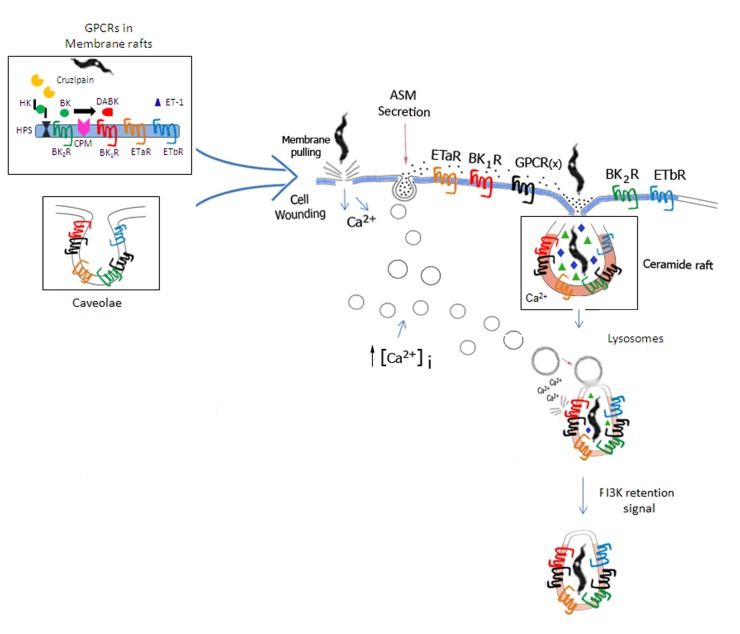
**Space filling model of GPCR-mediated invasion of cardiovascular cells.** Based on the unified model of *T. cruzi* invasion proposed by Fernandes and Andrews (2012), the scheme illustrates how GPCRs (including BKRs/ETRs) originally confined to lipid rafts or caveolae (left side of panel) might be incorporated into the molecular platforms that coalesce around ceramide-enriched vesicles generated by the housekeeping mechanisms of plasma membrane repair from cellular wounds. According to Fernandes and Andrews, extracellular calcium influx resulting from cell wounds (center of panel) trigger Ca^2+^-regulated lysosomal exocytosis, which in turn leads to the secretion of acid sphingomyelinase (ASM). Acting pericellularly, ASM generates ceramide-enriched endocytic vesicles. Next, BKRs, ETRs, along with several other GPCRs partners and several accessory signaling molecules translocate from lipid rafts/caveolae into the ceramide-platforms. In the resting state, signal transduction via BK_2_R/ET_A_R/ET_B_R (cross-talk) may likely depend on formation of homo or hetero-oligomers in multimolecular platforms coalescing in ceramide-enriched vesicles. During inflammation, the repertoire of GPCRs in the ceramide-rich endocytic vesicles is modified by the incorporation of inducible molecules, such as BK_1_R and CPM. ET-1 (host cell origin/autocrine route) and des-Arg-kinins (released from surface bound HK by cruzipain, then subsequently metabolized by CPM) bind to their respective GPCRs (ET_A_R/ET_B_R and BK_1_Rs). Feedback cycles might enhance the efficiency of the housekeeping plasma-membrane repair mechanisms via BKR/ETR-driven (i) induction of the canonical pathway of Ca^2+^-regulated lysosomal exocytosis and/or (ii) induction of the calcium-dependent lysosomal fusion with the nascent parasitophorous vacuole (Andrade and Andrews, 2004) and/or PIK3-dependent retention signals (Woolsey and Burleigh, 2004).

Further exploring the role of cruzipain as a virulent factor, Scharfstein et al. (2000) reported that activation of BK_2_R potentiated parasite uptake by non-phagocytic cells. Whether using CHO transfected cells, human umbilical vein endothelial cells (HUVECs), primary cultures of mouse (neonatal) cardiomyocytes (Todorov et al., 2003), or primary culture of human smooth muscle cells (HSMCs; Andrade et al., 2012), these studies indicated that the parasites relied on cruzipain activity to generate a [Ca^2+^]_i_-inducing signal (BK_2_R agonist) from HK displayed on host cell surfaces. Although the genetic ablation of the multicopy cruzipain genes remained a technical obstacle, experiments performed with membrane-permeable irreversible inhibitors of cruzipain and parasites overexpressing the cruzipain gene strongly suggested that the trypomastigotes critically depended on the enzymatic activity of cruzipain to stimulate parasite uptake by BK_2_R-positive target cells (Scharfstein et al., 2000). The authors hypothesized that upon trypomastigote attachment (posterior end) to host cell surfaces, the enzymatically active forms of cruzipain – localized in the flagellar pocket (Murta et al., 1990; Souto-Padrón et al., 1990) – rapidly diffuse into the secluded spaces formed by the juxtaposed host/parasite membranes (Scharfstein et al., 2000). Once confined to “synapses” (Tyler et al., 2005; Butler and Tyler, 2012), the active protease should be protected from targeting by natural inhibitors, such as cystatins, soluble forms of kininogens (Stoka et al., 1998), and α2-macroglobulin (Araújo-Jorge et al., 1994; Morrot et al., 1997) present in interstitial fluids. Considering that BK_2_R is sequestered to membrane rafts/caveolae (de Weerd and Leeb-Lundberg, 1997; Haasemann et al., 1998), we hypothesized that surface-bound HK (cruzipain substrate) may translocate in the plasma membrane before being delivered to the site of synapse (**Figure 3**), thus ensuring efficient kinin release/BK_2_R-mediated signal transduction (Scharfstein et al., 2000; Santos et al., 2006; Scharfstein and Andrade, 2011; Andrade et al., 2012).

While analyzing the outcome of infection in cultures of HUVECs (resting state), we found that BK_2_R did not promote parasite uptake in the absence of ACE inhibitors. This was not unexpected, because expression of ACE, a transmembrane metallopeptidase that potently degrades intact kinins, is strongly upregulated by endothelial cells. As predicted, BK_2_R-dependent parasite uptake by HUVECs is potentiated by ACE inhibitors (Scharfstein et al., 2000; Todorov et al., 2003; Andrade et al., 2012).

Noteworthy, ACE inhibitors were not essential for BK_2_R-driven parasite invasion of cardiomyocytes or smooth muscle cells (Todorov et al., 2003; Andrade et al., 2012). Although the levels of ACE were not measured, it is likely that these muscle cells express lower levels of ACE as compared to HUVECs. Along similar lines, TCTs induced IL-12 responses on splenic CD11c^+^ DCs via BK_2_R irrespective of presence/absence of ACE inhibitors (Monteiro et al., 2007). In summary, the outcome of Dm28c TCT interaction with cells that constitutively express BK_2_R is controlled by ACE in cell-specific manner.

## THE INDUCIBLE BK_1_R: A UBIQUITOUS GATEWAY FOR *T. cruzi* INVASION OF INFLAMED TISSUES?

A major challenge in Chagas disease research is to predict the clinical outcome of the chronic cardiomyopathy. About 30% of these patients develop a progressive form of cardiomyopathy characterized by the presence of diffused inflammation/fibrosis. As mentioned in the introduction, pathologists noticed the presence of microvascular abnormalities and altered ECM patterns in the heart of CCM patients (Higuchi et al., 1999). To our knowledge, BK_1_R expression was not systematically investigated in heart autopsy studies of chagasic patients despite the indications that dysregulated BK_1_R function might be detrimental to the heart in other disease models. For example, in animal models of STZ-induced diabetic cardiomyopathy (Westermann et al., 2009), BK_1_R-deficient mice showed attenuated cardiomyopathy as compared to wild-type mice, as evidenced by the decrease of cardiac inflammation, fibrosis, oxidative stress, and significant improvement of left ventricular function. Pertinent to studies linking heart remodeling to the ET up-regulation by parasitized cardiomyocytes (Tanowitz et al., 2005), there is indication that oxidative stress induced by ET-1 (ET_A_R-driven) and angiotensin I (AT_1_-driven) up-regulates BK_1_R, leading to activation of PI3K and MAPK in smooth muscle (Morand-Contant et al., 2010). Although not directly examined, it is conceivable that NF-κB-induction by proinflammatory cytokines secreted by antigen-specific intracardiac CD8^+^ T cells (Padilla et al., 2009; Silverio et al., 2012) might also up-regulate BK_1_R in the chagasic heart.

Todorov et al. (2003) were the first to demonstrate that Dm28 TCTs evoke interstitial edema via the sequential activation of BK_2_R/BK_1_R. In the same study, they highlighted the dichotomic nature of kinin signaling pathways: the parasites were able to invade activated target cells via the BK_1_R/CPM pathway (**Figure 1B**). Differently from results observed in resting HUVECs (which critically depend on ACE blockade to efficiently internalize TCTs via BK_2_R), the trypomastigotes invade lipopolysaccharide (LPS)-treated HUVECs through interdependent signaling (“cross-talk”) between BK_1_R and BK_2_R (Todorov et al., 2003). Notably, the “cross-talk” between BK_2_R and BK_1_R was also observed in invasion assays performed with primary mouse cardiomyocytes (Todorov et al., 2003), which are cell types that spontaneously express BK_1_R in culture systems.

Although studies with non-specific kininase I inhibitors have tentatively linked BK_1_R-dependent parasite uptake to the processing activity of these carboxypeptidases, the role of the transmembrane carboxypeptidase CPM was not directly addressed in the above studies. As mentioned in the introduction, BK_2_R agonists (BK/LBK) are converted into the BK_1_R agonists (i.e., [des-Arg]-BK/LBK) either by surface CPM or by a soluble (plasma-borne) CPM (**Figure 1A** and **Figure 2B**). Since the interaction medium used in our invasion assays was free of serum, it seemed unlikely that CPN was the processing enzyme critically involved in BK_1_R-driven parasite invasion of LPS-HUVECs or cardiomyocytes. Instead, we inferred that CPM is likely the enzyme generating the BK_1_R agonist that propels parasite invasion in our *in vitro* assays. Interestingly, Sangsree et al. (2003) studied the functional interplay between CPM and BK_1_R in great details in human lung microvascular cells activated by IL-1β and IFN-γ. First, they showed that BK_1_R signaling is responsible for the sustained NO response induced by intact BK (BK_2_R agonist), implying that CPM converted the intact BK into [des-Arg]-kinins. Using cells transfected with genes conding for CPM and BK_1_R, the authors disrupted lipid rafts with methyl-beta-cyclodextrin (MβCD). As predicted, this maneuver reduced the BK_1_R-dependent increase in [Ca^2+^]_i_ in response to stimulation with intact BK_2_R agonists, whereas addition of cholesterol rescued this BK_1_R-driven response. After showing that CPM and BK_1_R co-localized in lipid raft/caveolin-enriched membrane fractions (Zhang et al., 2008), they found that CPM/BK_1_R physically interact on the cell membrane, based on co-immunoprecipitation, cross-linking, and fluorescence resonance energy transfer analysis. In an elegant experiment using a novel fusion protein containing CPM at the N-terminus of the BK_1_R, Zhang et al. (2008) showed that these transfected cells were [Ca^2+^]_i_ responsive upon stimulation with intact kinins, but (as predicted) this response was no longer impaired by MβCD or by CPM antibody.

As already mentioned, in invasion assays performed with LPS-HUVECs or cardiomyocytes, we found that ACE inhibitors were not required to promote parasite uptake via BK_1_R, as opposed to the ACE inhibitor-dependent phenotype displayed by resting HUVECs (Todorov et al., 2003; Andrade et al., 2012). This implies that CPM-dependent generation of the BK_1_R is prioritized over the ACE-dependent pathway of BK/LBK degradation, hence consistent with the findings reported by Zhang et al. (2008). Since both CPM/BK_1_R and BK_1_R/BK_2_R are sequestered into lipid rafts and/or caveolae, it is conceivable that these GPCRs segregate together with HK (bound to heparan sulfate proteoglycans) into specialized plasma membrane microdomains (**Figure 3**). Of further interest, although BK_2_R and BK_1_R signal cells through fairly similar intracellular pathways, the regulation of inducible BK_1_R differs from BK_2_R in that the former is desensitized upon agonist binding only to a limited degree (Enquist et al., 2007).

## BKRs AND ETRs AS GATEWAYS FOR INVASION OF CARDIOVASCULAR CELLS: FUNCTIONAL LINK WITH THE SPHINGOMYELINASE-DEPENDENT PLASMA MEMBRANE PATHWAY?

In a previous section, we reviewed results of studies showing that Dm28c TCTs induce inflammatory edema through mechanisms involving cooperation between BKRs and ETRs (Andrade et al., 2012). To evaluate whether the parasites could exploit the ET pathway for invasion purposes, Andrade et al. (2012) studied the interaction of Dm28c TCTs with three types of host cells: HUVECs (which only express ET_B_R), primary cultures of HSMCs (which express ET_A_R and ET_B_R), and mouse neonatal cardiomyocytes (expressing both subtypes of ETRs). Using multiple pharmacological tools (subtype-specific ETR antagonists; neutralizing antibodies for each GPCR) and, in addition, interference RNA, Andrade et al. (2012) demonstrated that TCTs invade “resting” HUVECs via ET_B_R, while invasion of the muscle cells involved activation of both subtypes of ETRs. Notably, the combined treatment of the muscle cell cultures with ET_A_R and ET_B_R subtype-specific antagonists failed to decrease parasite infectivity of HSMC or cardiomyocytes over values induced by the individual drugs, thus recapitulating the “cross-talk” observed between BK_2_R/BK_1_R in cardiomyocytes or LPS-HUVECs (Todorov et al., 2003). Importantly, studies with CHO-ET_A_R or CHO-ET_B_R-transfected cells demonstrated that parasite invasion was efficiently blocked in subtype receptor specific manner, implying that formation of ET_A_R/ET_B_R heterodimers, “*per se*,” is not an absolute requirement for parasite entry. Since G-protein-coupled B_2_R and ETR compartmentalize in lipid rafts/caveolae (de Weerd and Leeb-Lundberg, 1997; Bremnes et al., 2000; Okamoto et al., 2000; Harada et al., 2002; Ostrom, 2002), we reasoned that in transfected mammalian cells the density levels of any particular GPCR (in lipid rafts/caveolae) might be well above those found in target host cell types that naturally overexpress these receptors (Andrade et al., 2012). If true, in natural muscle cells, the reduced proportion of any given subtype of GPCR may be compensated by cooperative interactions involving physical association with alternative GPCR “partners.” Assuming that ligand generation is not a limiting factor, this “space-filling model” predicts that GPCRs that might be present at higher density in such microdomains should have a better chance to coordinate signaling responses upon ligand binding, as seems to be the case for BK_2_R, ET_A_R, and ET_B_R (Andrade et al., 2012; **Figure 3**).

Although we did not prove that BK_2_R, ET_A_R, and ET_B_R form homo or hetero-oligomers of higher order in host cell attachment sites, our pharmacological studies support this possibility. Whether using subtype-specific GPCR antagonists, neutralizing antibodies, or iRNA interference (ET_A_R), our results showed that parasite invasion was inhibited roughly to the same extent. Furthermore, we did not observe additive effects by combining receptor subtype antagonists or iRNA approaches. These results suggested that pharmacological blockade of one GPCR partner is sufficient to dismantle the signaling function of entire unit, consequently reducing parasite internalization in 40–60% (Andrade et al., 2012).

Several years ago, it was demonstrated that cholesterol-depleting drugs reduce host cell susceptibility to *T. cruzi* infection (Fernandes et al., 2007). In a subsequent study, we explored the possibility that BKRs and ETRs compartmentalize in lipid rafts/caveolae, by treating HSMCs with MβCD (Andrade et al., 2012). As predicted, the cholesterol-depleting drug drastically reduced parasite (Dm28c) entry in HSMCs whereas addition of exogenous cholesterol to MβCD-HSMCs restored ET_A_R/ET_B_R/BK_2_R-dependent pathways of *T. cruzi* invasion. Although the physical association of these GPCRs was not demonstrated at the molecular level, these results suggest that the cross-talk between ETRs and BK_2_R may critically depend on the integrity of lipid rafts/caveolae (**Figure 3**). Interestingly, confocal microscopy studies performed with antibodies to anti-ET_A_R or anti-ET_B_R showed that parasite–cell interaction sites contained increased clusters of these GPCRs. These results are consistent with the concept that ET-1 and “kinins” activate their cognate GPCRs in lipid rafts/caveolae, perhaps translocated to synaptic sites (Scharfstein et al., 2000). As explained below, it is also possible that ET-1, ET precursors and HK (bound to heparan sulfate) exert their functional roles as agonists and/or precursors of GPCR ligands after their internalization in ceramide-enriched vesicles (**Figure 3**).

In a breakthrough, Fernandes et al. (2011) and Fernandes and Andrews (2012) elaborated a unified mechanistic concept for parasite invasion of non-phagocytic host cells which nicely embraced two seemingly distinct internalization routes (Andrade and Andrews, 2004; Woolsey and Burleigh, 2004), the first one driven by calcium-regulated lysosomal exocytosis, leading to plasma membrane fusion and a lysosomal-independent pathway involving parasite entry though nascent parasitophorous vacuoles. In this unified mechanism of infection, trypomastigotes invade host cells by subverting a housekeeping mechanism of repair from cell wounds which crucially depends on acid sphingomyelinase (ASM)-driven formation of ceramide-enriched endocytic vesicles. Triggered by the formation of stable lesions in the host plasma membrane, the repair response is initiated by the influx of extracellular calcium. This event in turn leads to calcium-regulated lysosomal exocytosis and ASM secretion to the extracellular environment. Further downstream, ASM cleaves the polar head group of sphingomyelin, generating ceramide. The repair process is terminated by the formation of endocytic vesicles, which then reseal the original plasma membrane lesions as they internalize. *T. cruzi* trypomastigotes are thought to inflict plasma membrane injuries upon adherence of the vibrant flagellum/cell body to the cell surface. Invasion “*per se*” occurs after the formation of ceramide-rich endocytic vesicles (Fernandes et al., 2011).

Although we have not provided direct evidence that BK_2_R, BK_1_R, ET_A_R, ET_B_R are translocated from lipid and/or caveolar microdomains into ceramide rafts of muscle cells (HSMC or cardiomyocytes) or HUVECs, this possibility deserves to be explored in light of evidence that ceramide-enriched microdomains spontaneously fuse to generate large macrodomains containing receptor clusters in multimolecular platforms (Jin et al., 2008). Using endothelial cells derived from coronary arteries, these authors reported that Fas-ligand driven activation of death receptor mediates the formation of redox signaling platforms in lipid rafts via ceramide production by ASM-driven hydrolysis of sphingomyelin. They also showed that formation of ceramide-enriched signaling platforms was canceled in endothelial cells treated with inhibitors of lysosomal function. In other words, the requirements for the formation of multimolecular signaling platforms in ceramide rafts of endothelial cells seem to recapitulate the requirements for parasite invasion via the house-keeping mechanisms of repair from cell wounds described by Fernandes and Andrews (2012). According our “space-filling model” (**Figure 3**), the signal transduction responses coordinated by these various GPCRs (e.g,., BK_2_R/ET_A_R/ET_B_R) may either occur (i) within the synaptic sites formed soon after adhesion and/or (ii) after translocation of these GPCRs into ceramide rafts, hence integrating the above mentioned multimolecular signaling platforms. Sufficiently flexible, this GPCR-dependent model of parasite internalization is compatible with linear or converging activation pathways. For example, the linear activation mode predicts that specific GPCR subtypes are differentially targeted to lipid rafts/caveolae or to ceramide-enriched vesicles and/or to nascent parasitophorous vacuoles (**Figure 3**). Once translocated to nascent parasitophorous vacuoles, some of these GPCR subtypes may generate parasite retention signals through PI3K-dependent remodelling of the actin cytoskeleton (Woolsey and Burleigh, 2004), and/or drive calcium-dependent lysosomal fusion to parasitophorous vacuole (Andrade and Andrews, 2004). Multiple contacts with the pathogen and signals transmitted by soluble agonists available at high levels in the edematous extracellular environment, such as ETs, BK, DABK, may then fuel parasite uptake *in vivo* through converging signaling pathways.

The possibility that these GPCRs might serve as gateways for *T. cruzi* infection of heart cells is worth exploring. According to Fernandes and Andrews (2012), *T. cruzi* propensity to invade muscle cells is dictated by the need of muscle cells to constantly repair sarcolemma injuries via the Ca^2+^-regulated pathway of lysosomal exocytosis and ASM-driven ceramide generation (McNeil and Steinhardt, 2003). Future studies may clarify whether the signals that promote parasite uptake by cardiomyocytes via interdependent signaling of (BK_2_R/ET_A_R/ET_B_R) may increase the efficiency of the house-keeping mechanisms of muscle cell repair from sarcolemma wounds. If confirmed, the convergence of house-keeping and GPCR-mediated signaling pathways may translate into mutual benefits to the host/parasite relationship, at least so during the indeterminate stage of chronic infection. Interestingly, the concept that BK_2_R signaling is protective to the heart tissues is supported by evidences that this pathway restores S2a-mediated sacroplasmatic Ca^2+^ uptake (Tschöpe et al., 2005; Spillmann et al., 2006). As chronic pathology evolves, perhaps the harmonious trade-off between parasite and cardiac muscle is gradually compromised owing to the ubiquitous up-regulation of BK_1_R by heart cells, including cardiomyocytes, cardiac fibroblasts, and inflammatory and/or TGF-β (regulatory/pro-fibrotic) macrophages. Flares of plasma leakage elicited by trypomastigotes occasionally released by from ruptured pseudocysts may propitiate the rise of extravascular levels of [des-Arg]-BK/LBK (BK_1_R agonists) and ET-1. Furthermore, the extravascular levels of ET-1 are further elevated as result of up-regulated expression of this the pro-oxidative mediator by parasitized cardiomyocytes (Tanowitz et al., 2005). A vicious cycle may be installed, favoring parasite entry in heart cells via the ET_A_R/ET_B_R/BK_1_R pathway at expense of increased inflammation and adverse cardiac remodeling (**Figure 2B**).

## HOST/PARASITE BALANCE, KKS AND THE PHENOTYPIC VARIABILITY OF *T. cruzi* SPECIES

The characterization of molecular determinants of pathogenic outcome is a major goal of basic and clinical research on CCM. Although the phenotype of the Dm28c may not be faithfully reproduced by every isolate of the highly polymorphic *T. cruzi* species, currently segregated into six DTUs (I–VI; Zingales et al., 2009), we may anticipate that comparative studies with other isolates should offer clues to understand the molecular determinant of pathogenicity of *T. cruzi*. For example, it will be interesting to know whether parasite strains belonging to particular DTUs share (or not) the ability to induce inflammatory edema through the cooperative activation of the TLR2/CXCR2/BKR_2_R/BK_1_R/ET_A_R/ET_B_R pathway. Although the expansion/contraction of this proteolytic cascade must certainly depend on additional parasite and host factors, the few players so far identified may help to investigate the phenotypic variability of natural *T. cruzi* populations. For example, strain-dependent variability on the expression levels of tGPI or, alternatively, in the extent of obscure mechanisms controlling shedding of lipid vesicles enriched with tGPI-linked mucins (Trocoli-Torrecilhas et al., 2009) might influence the trypomastigote ability to activate the KKS (Scharfstein and Andrade, 2011)

Alternative *T. cruzi* ligands are also known to activate innate sentinel cells through TLR4 (Oliveira et al., 2010), TLR9 (Bafica et al., 2006), or NOD1 (Silva et al., 2010), but it is presently unknown whether these pathways may interconnect to the KKS. Interestingly, Petersen et al. (2005) implicated TLR2 as the main upstream regulator of hypertrophy which *T. cruzi* trigger in isolated cardiomyocytes. On the other hand, *T. cruzi* strains that might express/shed higher levels of tGPI might somewhat blunt this potentially adverse phenotype by favoring low-grade release of cardioprotective kinins via cruzipain as described by Monteiro et al., 2006. In the chronic settings, the benefits of anti-apoptotic effects attributed to purified cruzipain (Aoki Mdel et al., 2006) and BK_2_R signaling (Chao et al., 2008) may be offset by the up-regulation of BK_1_R, a pathway that may synergize with TLR2 and ET_A_R, hence fueling inflammatory edema (**Figure 2**) and cardiac hypertrophy in chronically infected patients.

Another mechanism that may underlie the variable phenotype of *T. cruzi* strains is the variable expression profiles of cruzipain isoforms (Lima et al., 2001). For example, it is well established that cruzipain 2 (Dm28c strain) has narrow substrate specificity as compared to the major cruzipain isoform, i.e., the parasite kininogenase (Scharfstein, 2010). Predictably, strain-dependent variability in the ratio of expression between these two cruzipain isoforms may have impact on *T. cruzi* ability to invade host cells expressing BKRs (influence on tissue tropism) as well on its capacity to induce interstitial edema and T_H_1 responses via the kinin pathway. For similar reasons, we may predict that variations in the expression levels of chagasin, a tight-binding endogenous inhibitor of papain-like cysteine proteases – originally described in *T. cruzi* (Monteiro et al., 2001), may also influence the phenotype of *T. cruzi* strains. This possibility is supported by evidences (Aparício et al., 2004) indicating that TCTs of the G strain, which are poorly infective, display increased chagasin/cruzipain ratios as compared to Dm28c. Importantly, the infectivity of the G strain was enhanced upon addition of cruzipain-rich culture supernatants from Dm28 TCTs. In the same study, the authors pointed out that that vesicles shed by TCTs might serve as cruzipain substrates, presumably generating hitherto uncharacterized infection-promoting signals (Scharfstein and Lima, 2008). Hence, strain-dependent differences in the expression levels of tGPI and cruzipain isoforms may have impact on host/parasite relationship, either because kinins influence parasite infectivity, DC function and their ability to steer T_H_1-type effector development.

## CONCLUDING REMARKS

Host defense to infections depend on the mobilization of two distinct strategies in order to minimize infection-associated pathology: the first, innate immunity, is mobilized at the onset of infection with the purpose to eliminate or at least limit the spread of pathogens to the tissues (Medzhitov, 2009). The second strategy, involving products generated by proteolytic cascades and multiple endogenous “danger” signals emanating from injured tissues, has evolved to limit and repair the tissue damage inflicted by the microbial pathogen. The distinction between the mechanism underlying these overlapping processes in chronic parasitic infections, such as Chagas disease, is important because they profoundly affect the evolutionary dynamics of the of host–pathogen interactions (Rausher, 2001). Here we reviewed data showing that infective forms of *T. cruzi* can evoke interstitial edema through the activation of an inflammatory pathway initiated by one PRR (TLR2) and amplified through the “sequential” activation of the following GPCRs: CXCR2/BK_2_R/BK_1_R/ET_A_R/ET_B_R axis. Although presented as a linear cascade process for didactic reasons, this paradigm provides a useful framework to investigate the activation pathways that interconnect innate immunity to the KKS, a hub-like proteolytic network that generates proinflammatory kinin peptides while simultaneously activating three other proteolytic cascades, i.e., complement/coagulation/fibrinolytic systems – none of which were thus far explored in the context of CCM.

The evidence that activation of the kinin/BK_2_R pathway benefits the mammalian host emerged from our immunological studies (Monteiro et al., 2007). The analysis of the susceptible phenotype of BK_2_R-deficient mice (acute chagasic infection) demonstrated that DC sensing of the kinin “danger” signal via BK_2_R was essentially required for optimal generation of type 1 (host protective) effector T cells (Monteiro et al., 2007).

The dual role of the KKS in experimental Chagas disease is so far based on evidences that Dm28c trypomastigotes exploit BKRs/ETRs as (non-exclusive) gateways for cellular invasion of a broad range of non-phagocytic cells, including cardiomyocytes. We have proposed that trypomastigotes sharing the Dm28c phenotype might be able to transiently generate infection-promoting peptidergic ligands for GPCRs, such as BK and ET-1, in inflamed tissues through the induction of intersticial edema.

Interestingly, studies in other models of heart disease have associated KKS/BK_2_R signaling with protective cardiac functions, such as reducing apoptosis and chamber dilatation in the myocardium (Chao et al., 2008) and promoting restoration of S2a-mediated sarcoplasmatic Ca^2+^ uptake by cardiac cells (Tschöpe et al., 2005). Thus, it is tempting to speculate that BK_2_R signaling may bring about mutual benefits to the host/parasite equilibrium in the chagasic heart, perhaps during the quiescent phase (indeterminate) of chronic disease. Interestingly, there are a few reports describing beneficial effects of continuous ACE inhibitor treatment in animal models of Chagas disease (Leon et al., 2003; de Paula Costa et al., 2010). Although the clinical/pathological benefits of ACE inhibitors in CCM might be partially or totally ascribed to blockade in generation of pro-fibrotic angiotensin II, these results argue against an earlier proposition that ACE inhibitor-dependent up-regulation of the BK_2_R pathway might aggravate infection-associated pathology (Scharfstein et al., 2000).

In several models of sterile inflammation, the initial tissue damage is sufficient to initiate self-propagating biochemical and immunological processes with the aim to protect and rescue tissue integrity. Depending on the magnitude of the trauma, metabolic status, and host genetics, the collateral tissue damage associated with the activation of endogenous pathways of inflammation in the parasitized heart (Machado et al., 2000) may be extreme, causing irrevesible leison on cardiomyocytes. Studies *in vitro* have implicated TLR2 signaling in *T. cruzi*-induced pathways leading to cardiac hypertrophy (Petersen et al., 2005). It will be interesting to know whether the infection-associated damage of cardiomyocytes can be attenuated upon kinin release and BK_2_R signaling, perhaps reminiscent of some of the BK_2_R-dependent protective responses observed in ischemia reperfusion and diabetic cardiomyopathy (Messadi-Laribi et al., 2008; Tschöpe and Westermann, 2008).

Concerning BK_1_R, recent work in animal models of focal brain injury highlights the importance of therapeutic intervention on the KKS as a way to limit secondary damage caused by brain-barrier leakage (Raslan et al., 2010). As shown by these authors, pharmacological blockade of the inducible BK_1_R (but not BK_2_R) had remarkable protective effects on traumatic brain injury. Of interest in this context, we have previously reported that BK_1_R antagonist blunts the edematogenic response elicited by Dm28c TCTs in mice pretreated with LPS (Todorov et al., 2003).

Since chagasic patients display higher levels of ET-1 in the bloodstream (Petkova et al., 2000; Salomone et al., 2001), it is plausible that the edema resulting from pseudocyst rupture may promote the diffusion of blood-borne ET-1 through perivascular cardiac tissues. If true, these events may in turn trigger cardiac mast cell degranulation via the ET-1/ET_A_R axis, thus linking the ET pathway to the KKS (**Figure 2B**). Furthermore, considering that the expression of BK_1_R is up-regulated by the pro-oxidative ETs (Morand-Contant et al., 2010), and that ET-1 expression is robustly increased in parasitized cardiomyocytes (Tanowitz et al., 2005), it is conceivable that *T. cruzi* has evolved strategies to improve its infectivity at expense of increased inflammation, as recently shown in mice infected with the Y strain of *T. cruzi* (Paiva et al., 2012). Following a similar line of reasoning, we may predict that ET-1 and [des-Arg]-kinins, acting cooperatively, may propel parasite entry in cardiovascular cells through the signaling of BK_1_R, a subtype of GPCR that is ubiquitously up-regulated in inflamed tissues.

The sporadic formation of an intramyocardial edema in the proximity of an inflammatory lesion generated by pseudocyst rupture may alter tissue homeostasis, consequently modifying the dynamics of host/parasite interaction in this microenvironment. For example, the trypomastigotes navigating through intercellular cardiac spaces might be targeted by high-titered complement fixing/lytic antibodies (Krettli et al., 1982; Almeida et al., 1994) and/or by antibodies that inhibit host cell invasion (Schenkman et al., 1994; Tonelli et al., 2011). Induction of interstitial edema via activation of KKS might favor the rapid diffusion of these host protective antibody specificities into peripheral sites of infection. In addition, the inflammatory edema may allow the diffusion of antibodies against the C-terminal domain of cruzipain (GP25; Scharfstein et al., 1983). Antibody targeting of the immunodominant sulfated epitopes (Duschak and Couto, 2009) displayed on the C-terminal domain of cruzipain may either limit trypomastigote ability to efficiently invade cardiovascular cells (Meirelles et al., 1992; Scharfstein et al., 2000) and/or prevent anti-apoptotic responses which cruzipain otherwise induces in cardiomyocytes (Aoki Mdel et al., 2006).

For similar reasons, the heart conduction system might be targeted by auto-antibodies that are either specific for M2 muscarinic receptors (Medei et al., 2007) and/or β1-adrenergic receptors (Labovsky et al., 2007). Depending on their titers and fine-specificity, these auto-antibodies may evoke arrhythmia as a secondary consequence of plasma leakage elicited by trypomastigotes. If confirmed, this hypothesis may legitimate attempts to treat chagasic patients with drugs that target the ET/mast cell/KKS axis: by limiting leakage of auto-antibodies into the myocardium, these drugs may also mitigate cardiac arrhythmia.

Considering the long span of chronic infection, the myocardium of patients exhibiting deficient IL-10 production by macrophages or regulatory T cells (Costa et al., 2009) might be particularly prone to up-regulate the BK_1_R pathway in response to excessive collateral damage inflicted by pathogenic subsets of T cells. In this hypothetical scenario, the infiltrating type 1 effector CD8^+^ T cells may relentless fuel the pro-fibrotic pathways coordinated by BK_1_R/ETR. Of further interest, recent studies of sterile inflammation in transgenic mice subjected to cardiac pressure overload revealed that mitochondrial DNA (i.e., simulating symbiotic bacteria) leaks into autophagic vesicles of cardiomyocytes (Oka et al., 2012). Strikingly, the authors reported that the down-regulation of DNAse II, a lysosomal enzyme, fuels sterile inflammation in TLR9-dependent manner, provided that the mice are subjected to pressure overload (Oka et al., 2012). Although not explored in the context of CCM, it may not be surprising if chagasic heart debilitated by microvascular lesions, hypoxia, and immunopathology may be hyper-responsive to TLR9 signaling induced by *T. cruzi* mitochondrial DNA and/or self-DNA (from non-infected cardiomyocytes). If confirmed, it will be intriguing to know if BK_1_R and ETRs, acting cooperatively in the pro-oxidative environment that prevails in the chagasic heart (Dhiman and Garg, 2011) may up-regulate TLR9 and/or STING, another recently characterized sensor of self-DNA (Ahn et al., 2012). Ongoing studies in chagasic mice treated with BK_1_R blockers may clarify whether this therapeutic strategy may reduce parasite tissue load as well as myocardial fibrosis. Moreover, it is conceivable that BK_1_R antagonists may reduce chronic inflammation by preventing microvascular leakage and/or blocking parasite-induced recruitment of pathogenic subsets of anti-parasite CD8 T effector cells into the myocardium (Silverio et al., 2012), as reported in EAE (Dutra et al., 2011; Göbel et al., 2011).

In summary, our studies on the KKS have provided a general framework to investigate the intertwined proteolytic circuits that reciprocally couple anti-parasite immunity to inflammation and fibrosis in experimental CCM. Although limited to a single *T. cruzi* strain, Dm28c, the knowledge that emerged from 20 years of studies on this emerging field suggest that plasma leakage into the myocardium may shift the dynamics of host/parasite interaction in the inflamed heart through the activation of BKRs/ETRs. Hopefully, some of the lessons taken from this experimental work may help to elucidate the mechanisms underlying susceptibility to CCM, perhaps assisting efforts to improve the therapeutic management of chagasic patients.

## Conflict of Interest Statement

The authors declare that the research was conducted in the absence of any commercial or financial relationships that could be construed as a potential conflict of interest.
